# Functional Connectivity Density, Local Brain Spontaneous Activity, and Their Coupling Strengths in Patients With Borderline Personality Disorder

**DOI:** 10.3389/fpsyt.2018.00342

**Published:** 2018-07-27

**Authors:** Xiaoxia Lei, Yunjie Liao, Mingtian Zhong, Wanrong Peng, Qian Liu, Shuqiao Yao, Xiongzhao Zhu, Changlian Tan, Jinyao Yi

**Affiliations:** ^1^Medical Psychological Center, The Second Xiangya Hospital, Central South University, Changsha, China; ^2^Department of Radiology, The Third Xiangya Hospital, Central South University, Changsha, China; ^3^Center for Studies of Psychological Application, School of Psychology, South China Normal University, Guangzhou, China; ^4^Department of Radiology, The Second Xiangya Hospital, Central South University, Changsha, China

**Keywords:** borderline personality disorder, degree centrality, fractional amplitude of low frequency fluctuation, coupling strength, rs-fMRI

## Abstract

In this study, combining degree centrality (DC) and fractional amplitude of low frequency fluctuation (fALFF) analyses of resting state (rs)-functional magnetic resonance imaging (fMRI) data, we aimed to explore functional connectivity density, local brain spontaneous activity, and their coupling strengths in borderline personality disorder (BPD). Forty-three BPD patients and 39 demographically-matched controls underwent rs-fMRI after completing a series of psychological tests. Two-sample *t*-tests were performed to compare DC and fALFF between these two groups. Across-voxel correlation analysis was conducted to assess DC-fALFF coupling strengths in each group. Imaging parameters and psychological variables were correlated by Pearson correlation analysis in the BPD group. Altered DC and fALFF values in the BPD group, compared with the control group, were distributed mainly in default mode network (DMN), and DC-fALFF coupling strengths were decreased in the left middle temporal gyrus (MTG) and right precuneus in the BPD group. Additionally, insecure attachment scores correlated positively with left precuneus DC and negatively with fALFF of the right posterior cingulate cortex (PCC) in the BPD group. These altered DC and fALFF findings indicate that the BPD patients had disturbed functional connectivity density and local spontaneous activity in the DMN compared with control subjects. Their decreased connectivity-amplitude coupling suggests that the left MTG and right precuneus may be functional impairment hubs in BPD. Disturbed rs function in the left precuneus and right PCC might underlie insecure attachment in BPD.

## Introduction

Borderline personality disorder (BPD) is characterized by a pervasive pattern of emotional lability, impulsivity, interpersonal difficulties, identity disturbances, and disturbed cognition, as well as, in many cases, severe social impairment (1). Generally, signs of BPD emerge in late adolescence or early adulthood (2). BPD is one of the most common mental disorders, with an incidence of 2% in the general population, 10% among psychiatric outpatients, and up to 15–25% among psychiatric inpatients (2). Some 60% of BPD patients attempt suicide, with a lifetime suicide mortality rate of approximately 10% (3).

The causes and pathogenesis of BPD are not yet clear, but evidence has suggested that genetic, brain, environmental, and social factors may interact etiologically in the development of BPD (4, 5). BPD pathology has been related in recent years to a number of brain areas with altered structural or functional features (6–8). Structurally, voxel-based morphometric studies of patients with BPD have showed decreased gray matter volume (GMV) in the orbitofrontal cortex (9) and hippocampus-amygdala complex (10), and increased GMV in the precuneus (11) and posterior cingulate cortex (PCC) (8, 11). Functionally, task-based functional magnetic resonance imaging (fMRI) studies have indicated associations between brain activity and psychological processes in BPD (12). For example, decreased prefrontal regional activity and increased amygdala activity was associated with emotion dysfunction in BPD (13, 14). Activation of the insula and amygdala has been associated with interpersonal and social-cognitive deficits (15, 16). Additionally, functional connectivity aberrations in large-scale brain networks, including the central executive network (CEN), salience network (SN), and default mode network (DMN), have been related to emotional saliency and cognitive control in BPD (17). Altered functional connectivity within local networks has also been associated with BPD-related behaviors. For example, atypically low functional connectivity in the superior frontal gyrus has been found to be related to self-injurious behaviors and reduced pain sensitivity (18). Additionally, atypically high functional connectivity between the precuneus and frontal lobes has been found to be related to hyperactive processing of internal thoughts and self-referential information in BPD (19). Our research group has also found several BPD-related structural and functional brain anomalies, including enlarged GMVs of the right PCC and precuneus with resting-state (rs) brain hypoactivity (11, 20), disrupted white matter integrity and functional connectivity of the frontal medial cortex (20, 21), and augmented asymmetry and disturbed resting-state functional connectivity of the frontolimbic cortex (20, 22).

Over the last decade, rs-fMRI has become a widely-used neuroimaging modality for characterization of psychiatric and behavioral diagnoses, including major depressive disorder, obsessive-compulsive disorder, and conduct disorder. Rs-fMRI measures local cerebral deoxyhemoglobin level changes when subjects not performing a prescribed behavioral task, thus enables indirect assessment of spontaneous brain activities, and requires minimal cooperation from participants (just keep inactive) compared with task-related fMRI (23). However, there have been relatively few studies of rs functional alterations in BPD patients. Those that have been done mainly focused on large-scale networks and revealed altered functional connectivity in the DMN, SN, and CEN (17, 24). Kluetsch et al. found that BPD patients (all female cohort) had significant alterations in DMN connectivity during pain processing (25). In Doll et al.'s study, patients with BPD showed altered functional connectivity in the DMN, SN, and CEN, as well as their link with altered emotional saliency and cognitive control (17).

Nevertheless, the importance of specific brain network hubs remains unclear in BPD. Degree centrality (DC) and fractional amplitude of low frequency fluctuation (fALFF) are two fundamental parameters used for delineating network nodes and local characteristics of rs brain function (26). DC, a nodal graph-based metric, is defined as the number of functional connectivities between a voxel and the rest of voxels across the whole brain in a binary network, reflecting the importance of a node in a network (27). While fALFF can reflect target brain area functionality by tracking local neural signal amplitudes during resting state; it overcomes the challenge of interfering physiological noise that is associated with the traditional ALFF method by relating separate frequency bands to the total frequency range in ratios (28). DC and fALFF are diverse imaging analysis modalities, but both are closely related to brain metabolism in resting state. Hence, combining the two can provide a synergistic and more comprehensive approach to uncovering intrinsic brain network malfunctions (29, 30). Close coupling of these two parameters has been demonstrated under physiological conditions (31). Therefore, findings emergent from coupled DC and fALFF analyses may improve our ability to uncover mechanisms underlying physiological and pathological conditions (31).

In an earlier rs-fMRI study with multiple-algorithm analysis of spontaneous brain activity-associated ALFF, regional homogeneity of synchronized activity, and functional connectivity, we found evidence of alterations within the right PCC and adjacent precuneus in subjects with BPD during resting state, as well as indirect disturbances in the DMN (20). To build upon those findings, in this study, we used DC to further explore the functional connectivity density of brain regions, used fALFF to explore local spontaneous brain activity, and further examined sites with strong coupling between these two parameters in BPD. Our experiments were designed to test three hypotheses. Our first hypothesis was that, compared with control subjects, BPD patients would show altered functional connectivity density, local spontaneous activity, and imaging coupling strengths between DC and fALFF in resting state. Our second hypothesis was that imaging parameters (DC, fALFF) would correlate with psychological variables in the BPD patients. Finally, we hypothesized that the PCC and precuneus, identified in our aforementioned rs-fMRI study, would be confirmed as being altered in BPD.

## Subjects and methods

### Sample

A group of 43 young adults with BPD were recruited from outpatient clinics affiliated with the Second Xiangya Hospital of Central South University in Changsha, Hunan province, China. BPD was diagnosed independently by two qualified psychiatrists based on the structured clinical interview for axis II disorders (SCID-II) of the Diagnostic and Statistical Manual of Mental Disorders, Fourth Edition (DSM-IV). Every patient was administered the Structured Clinical Interview for DSM-IV Axis I disorders (SCID-I) to exclude past or current Axis I diagnoses (e.g., schizophrenia, delusional disorder, schizo-affective disorder, bipolar disorder). The presence of physical disorders with known psychiatric consequences (e.g., hypothyroidism, seizure disorder, brain injury) were also exclusionary.

A group of 39 age- and gender- matched control subjects were recruited by advertisements from the surrounding communities. The inclusion criteria for the control subjects were: no past or current history of any DSM-IV axis I or axis II disorders; no current medical problem; and no history of psychiatric disorders among first-degree relatives. They were assessed independently by two qualified psychiatrists using the SCID-I and SCID-II.

The study protocol was approved by the ethics committee of Central South University. Every participant signed an informed consent form at enrollment.

### Psychological assessments

Participants finished a battery of psychological tests prior to neuroimaging, including: the Short Affect Intensity Scale (SAIS), the Attachment Style Questionnaire (ASQ), the Childhood Trauma Questionnaire (CTQ), the center for Epidemiologic Studies Depression Scale (CES-D), and the State-Trait Anxiety Inventory (STAI). The SAIS was used to assess affect intensity; it includes three dimensions (positive intensity, negative affectivity, and serenity) (32). Only the negative affectivity score—which assesses negative emotional reactions such as nervousness, worry, sadness, and fear— was used (range, 1–6). The ASQ was used to assess attachment style; it includes five scales (confidence, discomfort, approval, preoccupied and secondary) (33). The confidence scale of the ASQ reflects secure attachment, and the other four scales represent insecure attachment. In this study, only the insecure attachment score was used (range, 32–192). The CTQ includes items related to sexual abuse, emotional abuse, emotional neglect, physical abuse, and physical neglect (score range, 28–140) (34). The CES-D and the STAI were used to assess participants' levels of depression and anxiety symptoms, respectively. The CES-D scale, STAI state anxiety subscale (SAI), and STAI trait anxiety subscale (TAI) scores ranged from 20 to 40 (35, 36).

### MRI data acquisition

All participants were scanned in a 3.0-T Philips Ingenia scanner. They were asked to relax, keep their eyes closed, and stay awake. Ear plugs and foam pads were used to minimize noise and head motion. Rs-fMRI images were obtained from gradient-echo-planar imaging sequence with the following parameters: TR (repetition time) = 2000 ms, TE (echo time) = 30 ms, flip angle = 90°, slices = 36, slice thickness = 4 mm, slice spacing = 4 mm, image matrix = 128 × 128, volume = 200, and volume interval = 2 s. Simultaneously, high-resolution T1-weighted structural images were collected from the three-dimensional magnetization prepared rapid acquisition gradient echo sequence (MPRAGE) with the following scanner parameters: TR = 7.44 ms, TE = 3.46 ms, flip angle = 8°, sagittal slices = 301, slice thickness = 1.2 mm, slice spacing = 0.6 mm, image matrix = 240 × 240, volume = 1, and voxel size = 1 × 1 × 1 mm^3^.

### Data preprocessing

Imaging data was analyzed in DPARSF (Data Processing Assistant for Resting-State fMRI software, http://rfmri.org/DPARSF) (37), based on Statistical Parametric Mapping 8 (SPM8, http://www.fil.ion.ucl.ac.uk/spm/) in a MATLAB platform (The MathWorks, Natick, MA). Data preprocessing included the following steps: (1) conversion of DICOM images to NIfTI files; (2) removal of the first 10 time points to minimize the influence of instability in the initial signals; (3) slice timing alignment, with the 18th slice set as the reference (due to continuous scanning); (4) spatial realignment to correct for head motion (subjects with head motion >2.0 mm or >2.0° were excluded); (5) registration of each subject's fMRI images to their segmented high-resolution T1-weighted anatomical images; (6) regression of nuisance variables, including white matter and cerebral spinal fluid signals; (7) normalization of fMRI images to standard Montreal Neurological Institute (MNI) templates with a resolution of 3 × 3 × 3 mm^3^; (8) smoothing with a 4-mm full-width-half-maximum Gaussian kernel; (9) linear detrending to discard physiological noise; (10) drift from scanner instabilities and head motion; (11) band-pass filtering (0.01–0.08 Hz).

### Calculation of DC and fALFF

DC, which is the number of functional connections of a given voxel, reflects voxel-wise whole brain functional connectivity strength, also known as functional connectivity density (38). Our DC calculations were derived from binary DC datasets and determined through Pearson correlations between the time course of a voxel and those of other voxels with a threshold of *r* > 0.25.

ALFF represents the amplitude of low frequency fluctuations (0.01–0.08 Hz) of each voxel in the whole brain, while fALFF is the ratio of the low-frequency (0.01–0.08 Hz) power spectrum to that of the entire frequency range (0–0.25 Hz) (28), both of which reflect brain spontaneous neuronal activity in resting state (39). Making use of the distinct characteristics of their signals in the frequency domain, fALFF has the advantage of providing enhanced sensitivity to neuronal activity while providing selective suppression of artifacts from non-specific brain areas and enhancing brain activity signals from cortical regions (28).

After preprocessing, the time series of each voxel was transformed to the frequency domain by fast Fourier transformation, and then the power spectrum of a given frequency band (0.01–0.08 Hz) and the entire frequency band (0–0.25 Hz) were then obtained. Considering the power of a given frequency is proportional to the square of the amplitude of this frequency component, the averaged square root was calculated across 0.01–0.08 Hz and 0–0.25 Hz bands. Then, each individual's fALFF value was acquired and transformed into a Z score (i.e., by subtracting global mean value and then dividing by the standard deviation) to allow further comparison between groups.

### Calculation of coupling strengths between DC and fALFF

We conducted across-voxel correlation analysis, which has been applied in many previous multimodality studies (40–42), to assess the DC-fALFF coupling strength. Correlation coefficients between the DC and fALFF time courses were used to depict coupling strengths. Across-voxel correlation analysis was also carried out at the level of individual subjects for the whole brain and within brain regions indicated to be altered by both DC and fALFF results.

### Statistical processes

Mean values are reported with standard errors of the mean (SEMs). Demographic data and psychological variables were compared between the BPD and control group in SPSS 20.0 (IBM). The processed fMRI data were further analyzed in REST software (43). Two-sample *t-*tests were performed to compare DC and fALFF values between the two groups, with age, gender, anxiety scores, and depression score as covariates. The results of our multiple comparison tests for DC and fALFF were corrected with the Rest AlphaSim program by combining the individual voxel *p* < 0.005 with cluster size >675 mm^3^ (equal to a significance level of *p* < 0.05) criteria.

Next, brain regions showing consistent group differences in DC and fALFF data were selected as regions of interest (ROIs). ROI signals and whole-brain signals (i.e., mean time course) were extracted for each subject in the REST program. Then, across-voxel correlation analysis between DC and fALFF was performed to acquire correlation coefficients (i.e., coupling strengths) for each group. Two-sample *t-*tests were performed to compare coupling strengths between the two groups in SPSS 20.0 (*p* < 0.05).

Finally, brain regions with significantly altered DC and fALFF values in the BPD group compared with control group were defined as ROIs. ROI signals from BPD patients were correlated to psychological variables by Pearson correlation analysis with significance level of *p* < 0.05.

## Results

### Demographics and psychological variables

The demographic and psychological characteristics of each group are summarized in Table 1. The BPD group and control group were similar in terms of age and gender. The BPD group had significantly higher scores than the control group in SAIS negative affection, ASQ insecure attachment, CTQ, CES-D, SAI, and TAI.

**Table 1:** Inter-group comparison of demographic and psychological variables.

**Variable**	**BPD group (*N* = 43)**	**Control group (*N* = 39)**	***t /x^2^*value**	***p***
**DEMOGRAPHICS**				
Age (years)	25.19 ± 3.06	24.79 ± 1.15	0.75	0.45
Gender (no. males/females)	22/21	16/23	0.85	0.36^a^
**PSYCHOLOGICAL ASSESSMENT SCORES**				
Negative affectivity	4.33 ± 0.83	3.48 ± 0.60	5.23	< 0.01
Insecure attachment	116.23 ± 18.56	96.47 ± 12.80	5.56	< 0.01
CTQ	37.39 ± 10.60	29.55 ± 4.02	4.35	< 0.01
CES-D	35.23 ± 6.91	27.44 ± 4.58	4.73	< 0.01
SAI	43.34 ± 4.70	44.04 ± 4.50	4.24	< 0.01
TAI	45.22 ± 4.38	42.42 ± 3.45	5.71	< 0.01

*CTQ, Childhood trauma questionnaire; CES-D, Epidemiologic Studies Depression Scale; SAI, State Anxiety Inventory; TAI, Trait Anxiety Inventory*.

a *Chi-square test; all other t-tests*.

### Inter-group comparison of DC and fALFF

**Figure 1:**
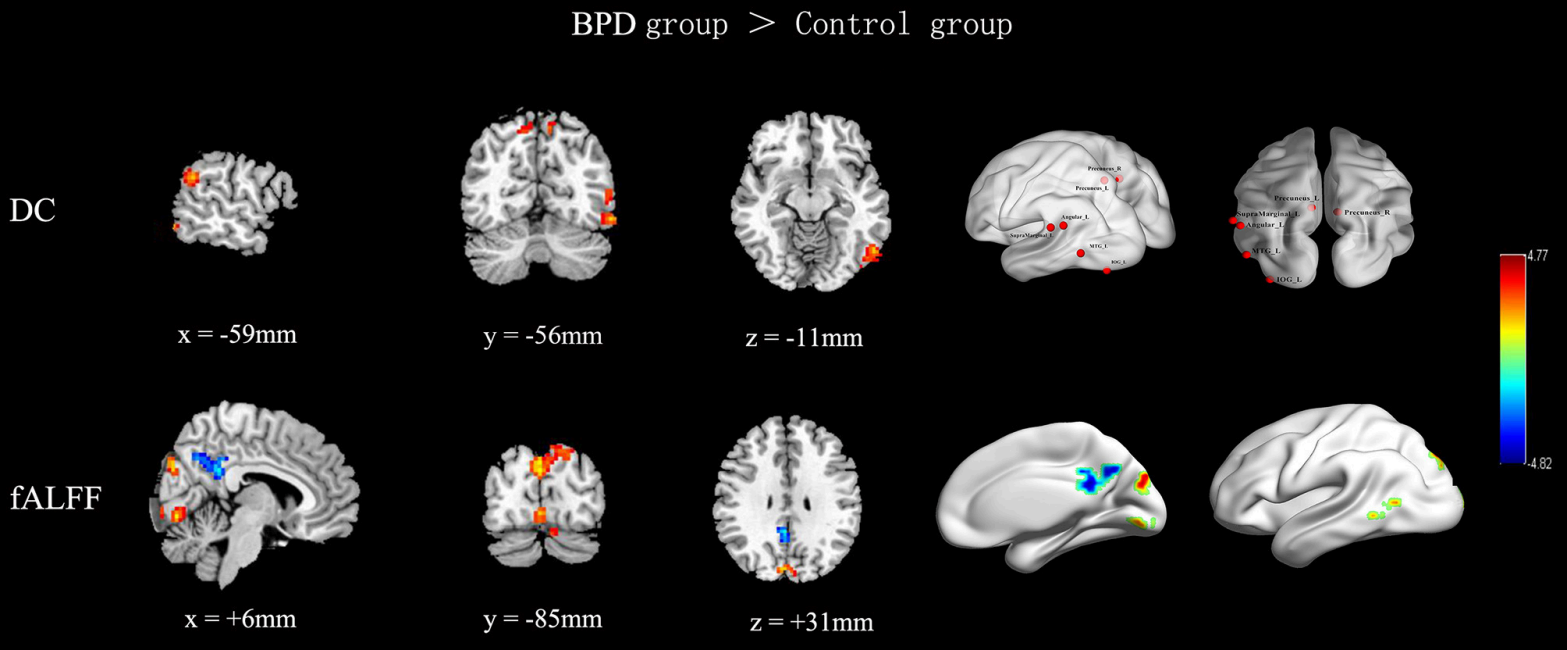
Inter-group comparisons of across-voxel correlation in the left MTG, right precuneus, and whole brain. Compared with the control group, the BPD group showed increased DC, mainly in the left MTG, left supramarginal gyrus and adjacent angular gyrus, left inferior occipital gyrus, and bilateral precuneus. Meanwhile, the BPD group showed decreased fALFF in the right PCC extending partly to the adjacent precuneus, and increased fALFF in the left MTG, right lingual gyrus, and bilateral cuneus. Results were corrected by AlphaSim (cluster *p* < 0.05, height threshold *p* < 0.005, and cluster size ≥675 mm^3^ for DC and fALFF). Color bar indicates T score scale.

The rs-fMRI parameter data for the BPD patients and control subjects are shown and compared in Figure 1 and Table 2. Note that, compared with the control group, the BPD group showed increased DC, mainly in the left middle temporal gyrus (MTG), the left supramarginal gyrus and adjacent angular gyrus, the left inferior occipital gyrus, and the bilateral precuneus, as well as decreased fALFF in the right PCC, partly extending to the adjacent precuneus, and increased fALFF in the left MTG, right lingual gyrus, and bilateral cuneus. Because two-sample *t*-tests showed that the left MTG in BPD subjects had consistently increased DC and fALFF, and the right precuneus reversely altered DC and fALFF, both regions were defined as ROIs. Time series (DC and fALFF values) for the left MTG, right precuneus, and whole brain were extracted to calculate DC-fALFF coupling strengths.

**Table 2:** Brain regions with significant group differences in DC and fALFF.

**Brain region**	**DC**			**fALFF**		
	**MNI coordinates (X, Y, Z)**	**Cluster size**	***t* value**	**MNI coordinates (X, Y, Z)**	**Cluster size**	***t* value**
MTG_L	−54, −69, 9	25	3.76	−54, −72, 9	31	3.7
Precuneus_R	15, −75, 48	41	3.74	5, −62, 38	42	−3.78
Precuneus_L	−9, −65, 58	37	3.52			
SupraMarginal_L Angular_L	−63, −48, 27	39	4.09			
IOG_L, MOG_L	−57, −62, −13	51	3.89			
PCC_R				6, −45, 27	33	−4.81
Cuneus_L				3, −84, 27	32	4.77
Cuneus_R				−5, −84, 34	29	4.3
Lingual_R				3, −81, −6	141	4.57

*Results corrected by AlphaSim. The clusters met p < 0.05 criteron, combining height threshold of p < 0.005 with a minimum cluster size >675 mm^3^. DC, degree centrality; fALFF, fractional amplitude of low frequency fluctuation; BPD, borderline personality disorder; MTG_L, left middle temporal gyrus; IOG_L, left inferior occipital gyrus; MOG_L, left middle occipital gyrus; PCC_R, right posterior cingulate cortex*.

### Comparison of coupling strengths between BPD group and control group

**Figure 2:**
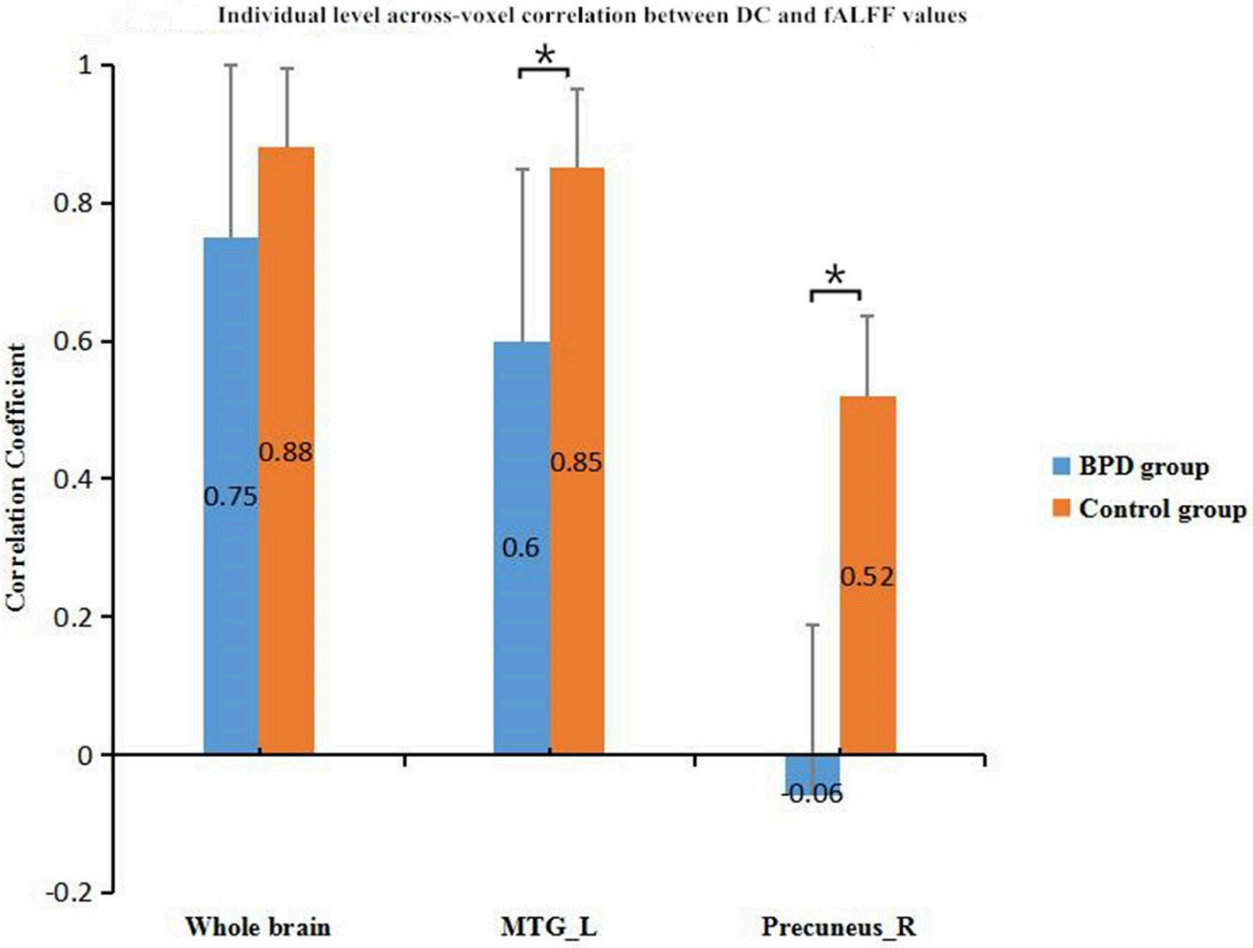
Inter-group comparison of across-voxel DC and fALFF correlations in left MTG, right precuneus, and whole brain. Coupling strengths in the left MTG and right precuneus were lower for BPD patients than controls. *p < 0.05; error bars are SEMs. fALFF, fractional amplitude of low frequency fluctuation; DC, degree centrality; BPD, borderline personality disorder; MTG_L, left middle temporal gyrus.

In across-voxel correlation analysis, BPD patients showed significantly decreased coupling strengths in the left MTG and right precuneus relative to control subjects (*p* < 0.05). They also had a decrease trend for the whole brain compared with the controls. Comparisons of the groups' correlation coefficients are provided in Figure 2.

### Pearson correlations between imaging parameters and psychological variables in the BPD group

**Figure 3:**
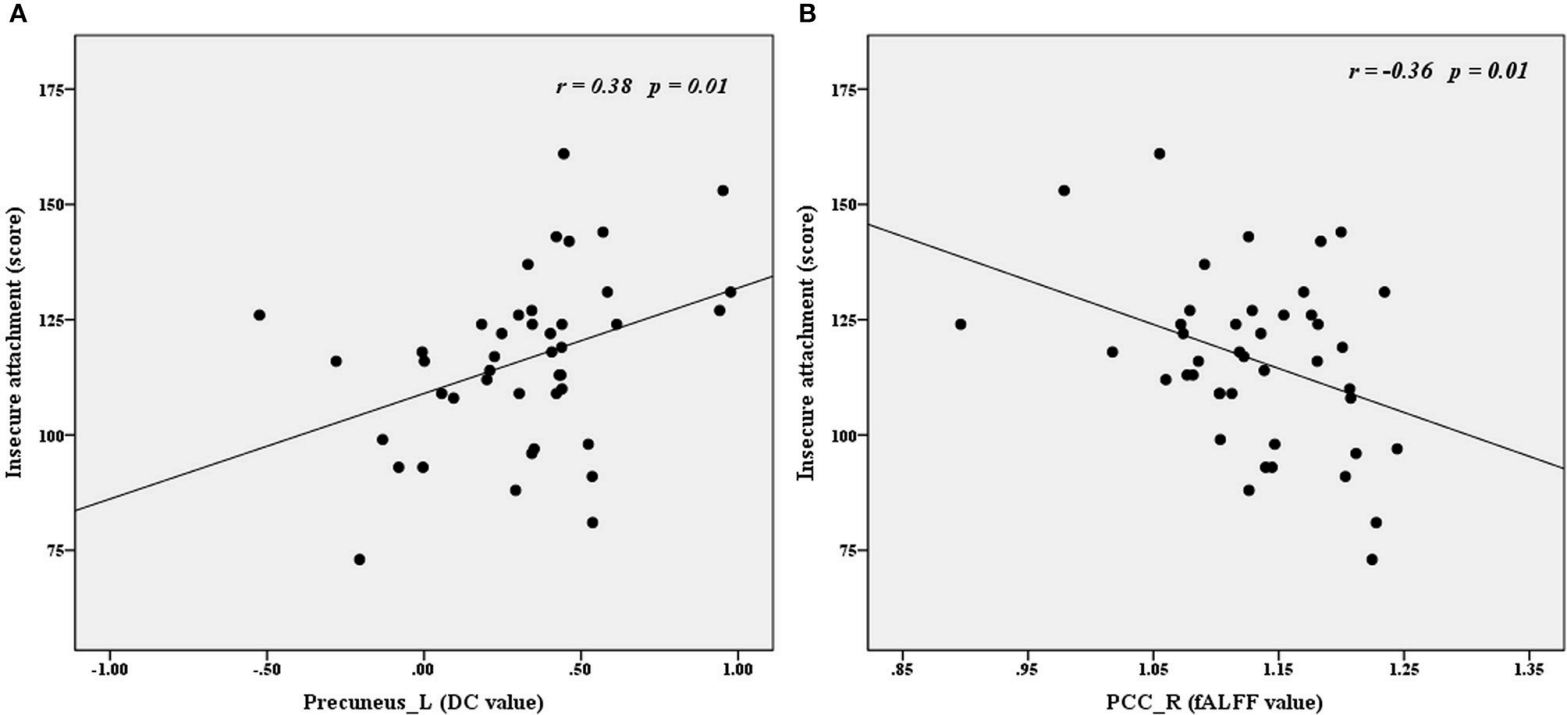
Correlations between imaging (DC and fALFF) and psychological variables in the BPD group. Insecure attachment scores correlated positively with left precuneus DC values **(A)** and correlated negatively with right PCC fALFF values **(B)**. fALFF, fractional amplitude of low frequency fluctuation; DC, degree centrality; Precuneus_L, left precuneus; PCC_R, right posterior cingutate cortex.

Time series (i.e., fALFF and DC values) were extracted to explore the correlations between imaging parameters and psychological variables in the BPD group. As shown in Figure 3, insecure attachment scores correlated positively with DC values for the left precuneus (*r* = 0.38, *p* < 0.01) and correlated negatively with fALFF values for the right PCC (*r* = −0.36, *p* < 0.05).

## Discussion

The results of the current study provide partial support for our hypotheses. We found that the brain regions with altered functional connectivity density and local spontaneous activity in BPD patients, relative to controls, were distributed mainly in the DMN. Meanwhile, DC-fALFF coupling strengths in the left MTG and right precuneus were significantly decreased in the BPD group compared with the control group. Pearson correlation analysis showed that insecure attachment scores correlated positively with left precuneus DC, and negatively with right PCC fALFF in the BPD group.

Functional brain activity in a task-free state, or rs, was demonstrated in rs-fMRI studies by Raichle et al., who attributed the active brain regions to the DMN, which included, originally, the medial prefrontal cortex, PCC, anterior cingulate cortex, precuneus, MTG, and inferior parietal lobule (44). Employing independent component analysis to explore rs functional brain networks in patients with BPD, Doll et al. further defined the main components of the DMN to include the superior frontal gyrus, inferior frontal gyrus, anterior cingulate cortex, PCC, precuneus, angular gyrus, and MTG, and also found altered functional connectivity in the DMN of BPD patients compared with that in healthy controls (17). In this study, using DC as a measure of functional connectivity density, which reflects the importance of specific network nodes (45), we found that, compared with the control group, the BPD group had increased DC in the left MTG, left supramarginal gyrus, adjacent angular gyrus, left inferior occipital gyrus, and bilateral precuneus, regions which are mostly components of DMN. These regions with increased DC could potentially be involved in transferring signals in a resting brain network in BPD patients, and suggest atypical DMN function in BPD.

Previously, Salvador et al. found reduced ALFF in the occipital lobes, right precuneus, and dorsal-PCC in BPD patients (46). In our earlier rs-fMRI study using ALFF, we found altered neural spontaneous activity, altered synchronization activity, and disturbed functional connectivity in the right PCC and precuneus of BPD patients (20). The fALFF method was introduced to enable comprehensive examination of local rs spontaneous brain activities, and has been proven to provide improved ALFF detection in rs-fMRI studies (28). Using this advantageous fALFF approach, we found that the BPD group had decreased fALFF in the right PCC, extending into the adjacent precuneus, and increased fALFF in the left MTG, right lingual gyrus, and bilateral cuneus, compared with control group, consistent with our and Salvador et al.'s previous work (20, 46). Together, these findings suggest that the brains of people with BPD have altered brain spontaneous activity in these areas. Given that the right PCC and precuneus are major structures of the DMN (28), which is related to self-referential processing, inner speech, emotional control, and episodic memory (47), we speculate that such altered local brain spontaneous activity in the DMN might be related to emotional lability and identity disturbances in BPD.

Across-voxel correlation analysis, which has been used to investigate associations among multiple-algorithm neuroimaging technologies, has the potential to help elucidate pathological mechanism with greater comprehensiveness and accuracy (31, 40, 41, 42). In this study, across-voxel correlation-based analyses demonstrated reduced DC-fALFF coupling strengths in the left MTG and right precuneus in BPD patients compared with the strengths in control subjects. Generally, BOLD frequency power is spatially segregated throughout the brain, and this segregation coincides to some extent with regional network architecture and connectivity (48). In other words, DC and fALFF tend to be closely coupled and similarly distributed. Diffuse reductions in connectivity-amplitude coupling in the left MTG and right precuneus in BPD suggests that these two areas might constitute functional impairment hubs in BPD.

In one Leibenluft's study about social attachment, when viewing pictures of one's love, participants would show stronger responses in brain areas associated with representing the mental states of others (theory of mind), namely, the anterior paracingulate, posterior superior temporal, and precuneus-posterior cingulate cortices (49). Jin et al. found that insecure attachment was negatively correlated with the GMV in the precuneus/middle cingulate cortex in young female adults (11). Minzenberg et al. found that the affective reaction to attachment was associated with temporal-limbic dysfunction in BPD patients (50). In the present study, we found that insecure attachment scores correlated negatively with fALFF values in the right PCC and correlated positively with DC values in the left precuneus. Along with previous studies, our results suggest that disturbed right PCC/left precuneus functions might underlie insecure attachment in BPD, while which needs further attachment task-related fMRI study to verify.

This study had several limitations. Firstly, though both DC and fALFF analyses are promising tools, more thoroughly developed behavioral indexes and fMRI paradigms with task activation should be employed to examine our hypotheses. Secondly, the relatively small sample size may limit the interpretation of our findings. Studies with larger sample sizes and more detailed neurocognitive domain assessments are needed to examine these results and to clarify neural mechanisms of BPD. Thirdly, because we found that some DMN structures had altered functional connective density, further studies are needed to examine potentially more global disruptions of the DMN. Lastly, further follow-up studies will help to clarify the causal relationship between insecure attachment and disturbed brain functions, in terms of functional connectivity density and local spontaneous brain activity, in BPD.

## Conclusion

In this rs-fMRI study, we found that DC-fALFF coupling strengths of the left MTG and right precuneus in BPD patients were significantly decreased compared with those in the control group. This diffuse decrease in connectivity-amplitude coupling suggests that these two brain areas may be functionally impaired hubs in BPD. The distribution of altered functional connectivity density and local spontaneous activity regions mainly within the DMN suggests indirect disturbance of functional connectivity in the DMN. The presently observed correlations of insecure attachment scores with left precuneus DC and right PCC fALFF suggests that disturbed resting state function in these regions might underlie the insecure attachment behavioral phenotype observed in BPD patients.

## Author contributions

XL was responsible for analyzing data and writing the original manuscript. YL and MZ collected the research data and wrote the manuscript. WP, QL, SY, XZ, and CT were responsible for data collection. JY was responsible for designing the study and revising the manuscript.

### Conflict of interest statement

The authors declare that the research was conducted in the absence of any commercial or financial relationships that could be construed as a potential conflict of interest.
